# Healthcare professionals’ views following implementation of risk stratification into a national breast cancer screening programme

**DOI:** 10.1186/s12885-022-10134-0

**Published:** 2022-10-12

**Authors:** Rachel Hawkins, Lorna McWilliams, Fiona Ulph, D Gareth Evans, David P French

**Affiliations:** 1 The Christie NHS Foundation Trust, Wilmslow Rd, Manchester, M20 4BX UK; 2 Manchester Centre for Health Psychology, Division of Psychology & Mental Health, School of Health Sciences, Faculty of Biology, Medicine and Health, University of Manchester, Manchester, UK; 3 NIHR Manchester Biomedical Research Centre, Manchester Academic Health Science Centre, Manchester University Hospitals NHS Foundation Trust, Manchester, England; 4 Nightingale & Prevent Breast Cancer Research Unit, Manchester University NHS Foundation Trust, Southmoor Road, M23 9LT Wythenshawe, Manchester, UK; 5 Department of Genomic Medicine, Division of Evolution and Genomic Science, Manchester Academic Health Science Centre, University of Manchester, Manchester University NHS Foundation Trust, Oxford Road, M13 9WL Manchester, UK

**Keywords:** Risk-based screening, Implementation, Healthcare professionals, Qualitative research, Risk-stratification, Breast cancer

## Abstract

**Background:**

It is crucial to determine feasibility of risk-stratified screening to facilitate successful implementation. We introduced risk-stratification (BC-Predict) into the NHS Breast Screening Programme (NHSBSP) at three screening sites in north-west England from 2019 to 2021. The present study investigated the views of healthcare professionals (HCPs) on acceptability, barriers, and facilitators of the BC-Predict intervention and on the wider implementation of risk-based screening after BC-Predict was implemented in their screening site.

**Methods:**

Fourteen semi-structured interviews were conducted with HCPs working across the breast screening pathway at three NHSBSP sites that implemented BC-Predict. Thematic analysis interpreted the data.

**Results:**

Three pre-decided themes were produced. *(1) Acceptability of risk-based screening*: risk-stratification was perceived as a beneficial step for both services and women. HCPs across the pathway reported low burden of running the BC-Predict trial on routine tasks, but with some residual concerns; (2) *Barriers to implementation*: comprised capacity constraints of services including the inadequacy of current IT systems to manage women with different risk profiles and, (3) *Facilitators to implementation*: included the continuation of stakeholder consultation across the pathway to inform implementation and need for dedicated risk screening admin staff, a push for mammography staff recruitment and guidance for screening services. Telephone helplines, integrating primary care, and supporting access for all language needs was emphasised.

**Conclusion:**

Risk-stratified breast screening was viewed as a progressive step providing it does not worsen inequalities for women. Implementation of risk-stratified breast screening requires staff to be reassured that there will be systems in place to support implementation and that it will not further burden their workload. Next steps require a comprehensive assessment of the resource needed for risk-stratification versus current resource availability, upgrades to screening IT and building screening infrastructure. The role of primary care needs to be determined. Simplification and clarification of risk-based screening pathways is needed to support HCPs agency and facilitate implementation. Forthcoming evidence from ongoing randomised controlled trials assessing effectiveness of breast cancer risk-stratification will also determine implementation.

**Supplementary Information:**

The online version contains supplementary material available at 10.1186/s12885-022-10134-0.

## Background

Regular breast screening detects cancers at an earlier stage, allowing earlier intervention and reducing breast cancer mortality[1]. There is continuing debate regarding the balance of these benefits versus the harms of breast cancer screening, notably over-diagnosis and false-positive test results[1, 2]. Personalised risk-stratified breast screening has the potential to improve this balance[3, 4]. Risk algorithms enable individual estimates of cancer risk to be calculated based on a combination of risk factors, and have been shown to perform well in an English screening population[5]. These algorithms allow tailored prevention and early detection for women at higher risk[6] and possibly reduced screening for women at lower risk [7]. With no randomised controlled trial (RCT) evidence to date[8], the effectiveness of risk-stratified screening at preventing the development of higher-stage breast cancers is being tested internationally in the WISDOM and MyPeBS trials[9, 10].

As well as demonstrating effectiveness, before implementation of risk-stratified screening, it is crucial to understand issues which affect the feasibility of this[11]. The BC-Predict study aimed to ascertain the feasibility of implementing risk-stratified screening via the NHS Breast Screening Programme (NHSBSP). BC-Predict recruited at three screening sites in north-west England (August 2019–June 2021). Thousands of women were offered risk stratification in BC-Predict, provided data on risk factors, and received personalised 10-year risk estimates after receiving negative screening test results. Higher risk women have been offered risk-consultations, chemoprevention, and increased mammogram frequency where appropriate[12].

A key element of feasibility of risk-stratified screening is whether it is acceptable to healthcare professionals (HCPs)[13]. Previous research on acceptability of risk-stratified screening has primarily collected data prior to implementation or considered this in hypothetical terms[11, 14, 15]. Focus groups with breast screening site HCPs conducted prior to implementing BC-Predict concluded that personalised risk-screening is a positive step but identified concerns regarding service capacity and additional workload. These issues have also been echoed in other studies[14, 15], as well as concerns with risk communication to women[11, 15, 16]. Some of this work fed into the development of care pathways and operational procedures for BC-Predict, to make the processes as user-friendly as possible[15].

The present study interviewed HCPs following the completion of BC-Predict. The aims of this study were to investigate HCP views on the acceptability of BC-Predict, on implementing risk-based screening and to explore barriers and facilitators to the wider implementation of risk-based screening generally.

## Methods

### Design

A cross-sectional qualitative design with semi-structured telephone interviews was used in this study to explore HCPs experiences of the BC-Predict study (see appendix A in supplementary material for Consolidated Criteria for Reporting Qualitative Research)[17].

### Setting and participants

Participants were eligible for the present study if they were a HCP who worked at a BC-Predict NHSBSP (including Family History Risk and Prevention Clinics; FHRPC) site. One site conducted all risk-consultations. Individual study invites and participant information were circulated via email, or in person by study staff, to all staff in the following professional groups: radiology, breast screening management/administration, radiography and FHRPCs (including breast care nurses). Additionally, nineteen staff at associated primary care practices (where at least one BC-Predict participant was registered) were invited by post. Those interested contacted the study team via email to arrange an interview.

Fourteen HCPs working across the three NHSBSP sites took part in a telephone interview (October 2021-April 2022). See Table 1 for participant occupation. Some participants took part in up to two previous focus group studies prior to implementation of BC-Predict[11, 16]. No primary care practice responded to study invitations.

**Table 1:** Occupation of study participants

Healthcare Profession	Participants (n = 14 total)
Screening office manager	3
Screening programme manager	2
Nurse (family history clinic)	2
Doctor (family history clinic)	2
Doctor/consultant (radiologist)	2
Mammographer/radiographer	3

### Data Collection

Audio-recorded, semi-structured individual telephone interviews (lasting on average 40 min; range 24–60 min) were guided by an interview schedule. Audio-recorded verbal consent was obtained prior to any study procedures. The interview schedule consisted of two sections to explore experiences of running BC-Predict and views on implementing risk-stratified screening nationally (see appendix B in supplementary material). This was iteratively developed (FU, LMW) and reviewed by a clinical geneticist, health economist, health psychologist and medical oncologist.

Interviews were conducted by two female researchers (RH, MSc and LMW, PhD) with a psychology disciplinary background, postgraduate training and experience conducting qualitative research with HCPs. Both researchers had no previous involvement in the pre-implementation focus group study, removing assumptions relating to the research topic. Participants had no prior relationship with the researchers who conducted their interview. Participants were either at home or in their workplace during the interview. All participants were provided with a flow chart figure of the pathway which they could refer to during the interview (a version of which is attached to supplementary file). Following each interview, field notes were documented, capturing key points in each interview to potentially probe further in subsequent interviews and to discuss this in relation to data sufficiency[18]. Data collection continued until all possible participants had been invited twice and the study team were satisfied sufficient data had been collected to answer the research question[19].

### Analysis

Interviews were transcribed and analysed by thematic analysis in NVivo 12[20]. This enables an in-depth insight into the experiences and perspective of HCPs working in applied health settings, and provides a robust method of qualitative analysis presented in a readily accessible manner[21]. Data were coded and analysed using a realist perspective[22, 23] with data viewed as a true representation of participant views and experiences.

Given the focus on acceptability, barriers, and facilitators to implementation of risk-stratified breast screening, an a-priori approach was taken driven by the research question. Themes were pre-decided by the research team and codes driven by the data. RH and LMW double coded one transcript as part of data familiarisation. Subsequent transcripts were coded by RH, with support from LMW, FU and DF. First, audio-recordings were listened to, and transcripts repeatedly read line-by-line by RH. Conducted at the inductive-manifest level, codes were data driven by participants’ accounts, which represented their views and experiences of implementing BC-Predict. Throughout this process, RH, LMW, FU and DF met to discuss codes and to develop the coding and theme structure and explanations within the data. Repetitive coding and refinements were conducted until a final thematic structure was agreed by the research team.

## Results

Coding was organised under three themes: (1) Acceptability of risk-based screening, (2) Barriers to implementation, and (3) Facilitators to implementation.

### Theme 1: acceptability of risk-based screening

#### Encourages women to take mitigating action

A strong consensus across sites and professions was that implementing risk-stratified screening was a positive step for the next generation of breast screening. An individualised risk assessment was perceived as more logical than providing identical screening for all women regardless of risk.

*“…there’s a good sort of foundation to run a service that offers different elements to a patient so it’s not just having the mammogram, it’s about looking at it holistically. I think if we were able to embrace things a bit like that more, then we’d be providing a better service…” (HCP0011; Mammographer/Radiographer)*.

Participants across the pathway and sites perceived risk-stratified breast screening as beneficial for women attending screening, by identifying those who may otherwise be unaware of their risk and providing them with information and services to manage their risk.

*“It is good picking up women who haven’t really accessed the service via their GP, who did have quite a prevalent family history. Then it’s a good avenue to pick up those women and bring them in and offer them a higher screening service.” (HCP010;* FHRPC *Nurse)*.

Participants discussed how risk-stratification enables agency by offering women different options to make decisions about risk reduction:

*“It’s going the next level, it’s saying, we know what the risk factors are but here’s what we’re going to do about it, we’re going to offer these women some tangible benefits to help reduce their risk or improve the chances of early detection.” (HCP008;* FHRPC *Doctor)*.

It appeared a strong consensus across participants that the concept of risk-stratification was largely positive; HCPs valued the benefit to both services and women in implementing this approach.

#### Low burden to routine tasks

Whilst screening office managers and radiographers across sites described challenges of setting up the software for BC-Predict, this was a short-term obstacle. Following study set-up, participants across the pathway and sites highlighted the minimal impact on their workload:

*“Once it was set up, it seemed to, run from our perspective, it ran quite well. There didn’t seem to be many hiccups in terms of the technical aspects. To be honest, from my personal perspective, I didn’t really notice it.” (HCP013; Screening Office Manager)*.

This was echoed by radiographers/mammographers and those responsible for risk-consultations, who reported minimal impact on appointments and tasks similar or the same as current roles:

*“…in terms of our mammogram itself, I don’t think it alters it at all. It was part of the interview process that we do with every patient anyway and there’s always going to be extra questions of whatever nature that you’d have to deal with.” (HCP011; Mammographer/radiographer manager)*.

When considering national implementation, some participants perceived minimal burden on workload but only if mammogram and risk consultation formats remain unchanged.

#### Ethical considerations: anxiety and screening disparities

HCPs across the pathway at the site conducting risk-consultations discussed how risk-stratification could potentially alleviate or worsen anxiety for women, but this was not seen as sufficient grounds to advise against implementation of risk-stratification. Participants at this site discussed how women informed of being moderate or high-risk would naturally feel more anxious, particularly if this risk result was not anticipated by the individual. However, having a secure pathway for high-risk women was perceived by one participant to minimise any anxiety induced in higher risk women:

*“I think it’s quite clear that, yes, they may be made aware that they are at higher risk. However, they’re not just told they’re at higher risk and then, sort of, left to deal with it. You know, they have, like, a clear pathway of what’s going to happen because I think that should reassure them because it reassures us” (HCP003; Screening Office Manager)*.

Participants also discussed potential NHSBSP disengagement from women. Due to a complicated risk-stratified process, women may be left feeling overwhelmed with information. Additional concerns from FHRPC staff and a radiographer highlighted that women might subsequently opt for private healthcare if unable to access genetic testing or enhanced screening at their screening site:

*“We can’t do MRI screening for every woman with high breast density. But you could consider to have that done privately. But then, that’s not very fair for the women who can’t afford private health care.” (HCP010;* FHRPC *Nurse)*.

The potential alleviation or increase of anxiety for women was also perceived as dependant on the individual’s pre-existing level of anxiety or worry. Participants thus highlighted ensuring implementation does not exacerbate service and health inequalities for women accessing risk-based screening.

### Theme 2: barriers to implementation

#### Managing additional demand

An anticipated increase of women into screening and capability of services to manage this demand was perceived a barrier, exacerbated by current increased demand, such as recovering round-length due to COVID-19.

*“The demand on the service is already I think at a point where we’re struggling to stay on top. So I think anything added would just increase the pressures” (HCP007; mammographer/radiographer).*

Across the pathway, participants expressed concerns regarding the scale of high-risk women accessing additional screening, even if numbers were small. Additional reporting of images appeared problematic for radiologists and screening managers at two sites, particularly if the time to download the images onto a risk-based screening system significantly added to their reporting time.

*“It just depends on how long it takes for each of these things to get uploaded into something and downloaded into something, and then for us to evaluate it and put our report in…if it’s going double that or triple that there will be a significant increase overall in the reporting times and it will impact on staff.” (HCP009;Radiologist)*.

In addition, one FHRPC doctor experienced doubled clinic capacity accounting for BC-Predict during the study.

*“Even it was only another five per cent of women that needed to be having annual mammograms, that’s still quite an increase.” (HCP008;* FHRPC *Doctor)*.

When questioned on implementation, different HCPs reported workforce shortages and high mammography staff turnover exasperating concerns to manage additional screening and reporting, and for staff to manage a possible influx of queries from women. This coupled with the national misalignment of resources between different sites (equipment, departmental structure, genetic testing, and chemoprevention knowledge), meant implementation demands will be unique to each site:

*“A lot of family history nurses are actually breast care nurses that have their symptomatic workload and their risk assessment of family history is added on to their other workload. […] the service is very different in different parts of the country.” (HCP009; Radiologist)*.

In line with this, funding for staff and screening infrastructure, and for equipment was viewed by participants at all sites as a pre-requisite across the pathway.

#### Clarity requirements to implement nationally

When prompted about the pathway, participants were unclear how women risk-stratified to low, medium, or high-risk would align with future screening pathways and FHRPC. This was especially true for those sites operating mobile screening vans as these set locations were dependent on the screening interval and invited population.

*“It seems a lot more complicated once you do the risk stratification if they go into like, say, a one-year pathway, a five-year pathway, a three-year pathway.” (HCP014; Radiologist).*

Participants across sites were unclear if high-risk women identified through risk-stratification would be referred in to a local, national, or new service. Radiologists at one site alluded to potential risks for women potentially missing their screening or being invited at the wrong time. An additional layer of complexity was highlighted with contradictory NHSBSP and national surveillance guidance for high-risk/very high-risk women and how that might impact women referred from different areas.

*“There’s a national breast screening, which this is involved in, but then we’ve got local… We’ve already got very high risk, which are the genes and everything but where would these women fit? (HCP001; Screening Office Manager)*

Participants from FHRPCs outlined staff and structural differences across the country which impact on high-risk referrals through this pathway.

*“Most family history clinics would tend to want to do their own risk assessment from a referral and it, whether that counteracts what the BC-Predict has given to that patient, that can create confusion.” (HCP010;* FHRPC *Nurse)*.

Simplifying and standardising the pathway that aligned within the NHSBSP and supported equal screening opportunities for women was perceived as a logical step by many participants. Participants in FHRPCs identified the need for protocols to support this.

#### Inadequate screening software and hardware

Despite all UK screening services using the same software, this was described by many participants as antiquated and lacking the ability to support risk-stratified screening:

*“It just seems really complicated and a bit of a concern that the technology and the software can’t really cope with that, and we could end up, you know, with incidents because we don’t actually invite everybody correctly.” (HCP014; Radiologist)*.

During BC-Predict, unanticipated software setup challenges imposed additional workload for sites, particularly in obtaining images for mammographic density from mobile units:

*“There were all sorts of implications here and there that we maybe hadn’t realised would be such an issue. What with the aerial, extending the height… They did get them eventually, but it was probably a bit more of a longer, more complex process than we probably first anticipated. (HCP013; Screening Office Manager)*

It appeared the lack of an integrated screening software to safely gather risk information and the complexity of screening invite systems was a prominent concern across participants, highlighting constraints of the present software used across breast screening.

### Theme 2: facilitators to implementation

#### Stakeholder consultation

Multidisciplinary conversations across all HCPs and with the national team were viewed as vital for coordinating national implementation of risk-stratified screening. Focus groups run to elicit challenges prior to BC-Predict implementation were viewed as a good opportunity to ask questions:

*“I think the focus groups done at the beginning were a really, really good idea. They gave everybody involved the opportunity to, sort of, come up with queries and questions and ideas…so I’d say do that with every, sort of, programme that you go into.” (HCP003; Screening Office Manager)*.

Taking a similar approach, by engaging all stakeholders across the pathway and ensuring dialogue with all individuals, was viewed as a facilitator for national implementation:

*“I think there has to be a huge sort of consultation with the service, the people that…all the stakeholders really to start with, just to understand what the practical issues may be.”(HCP012; Mammographer Radiographer Manager)*.

Participants with a variety of professional roles highlighted the importance of ensuring smaller sites are involved in subsequent consultation about this.

#### Supporting staff and training needs

Participants suggested how implementation could be supported, drawing upon current and future resource and training needs. Across all different professional groups, there was enthusiasm for risk-screening administration staff to oversee the programme and in particular *“dedicated staff members to just look after the higher risk screening ladies.” (HCP006; Screening Programme Manager).* Retention and recruitment of mammography staff was perceived as important and suggested by all radiographers. First, defining the workload and resource required across the pathway would be necessary:

*“if that’s the way forward, they would have to look at how many x-ray machines you have and how many staff you have, if they’re able to be increased in mammograms” (HCP006; Screening Office Manager)*.

When participants were prompted on training needs, guidance for screening offices and mammographers to support women’s queries was identified. This included the hope for alignment of NHSBSP and high-risk screening guidance. Medical guidance for nurse specialists and primary care was also suggested including how to complete the risk questionnaire.

*“With the chemoprevention aspect of it, which isn’t something that we get involved with at the moment, like a medicines medical point of view. So, I’m guessing there would have to be either some nursing involvement or some kind of medical guidelines or guidance for that” (HCP004; Screening Programme Manager)*.

HCPs identified possible solutions to support national implementation and key next steps including identifying and quantifying the additional need that will be required.

#### Supporting equitable access

Tools to support women’s information access was viewed as key for implementation. In BC-Predict, queries were managed by the study team by telephone and email. Screening programme managers and radiographers across sites, including a site who discussed the high ethnic diversity in their local population, viewed this a useful tool for women and for implementation. To ensure minimal impact on mammogram appointments, telephone helplines and information packs were suggested so this is available prior to appointments.

*“Yes, some sort of helpline or just somebody to just ask a query. Maybe an online sort of forum, maybe. Some information that you could just go simply to a portal and just type a question in but I think a phone line’s probably the best thing from our local area point of view. (HCP012; Mammographer/Radiographer Manager)*

Participants working at a site with a highly ethnically diverse population explained challenges with screening uptake in which the importance of communicating about risk-stratified screening in other languages was highlighted. Participants at other sites also discussed the need for standardised and simple information on risk-stratification

“*So you have to provide the same information to every woman” (HCP010;* FHRPC *Nurse).*

*“We need to be able to communicate the study to all the women that are eligible for breast screening within our local area and not just the English-speaking ones or English…it’s more the English reading” (HCP012; Mammographer/Radiographer Manager)*.

To support women’s access, general practitioners (GPs) were suggested by different participants working across FHRPCs to support amalgamating risk factors and by also collecting risk factors at GP consultations that could be shared with the NHSBSP at the point of mammogram referral. GPs were also suggested for supporting risk-screening referrals, and aiding women’s decision-making. However, capacity and capability of GPs to support this was a consideration for implementation.

*“Looking at all the risk factors. Some of this stuff would be readily accessible to the GP practice, even though it’s just maybe a question worth asking, whether or not it’s something that they could quickly facilitate” (HCP013; Screening Office Manager)*.

Ensuring risk-stratified screening meets the needs of all women across ethnically and culturally diverse communities was therefore a priority for these participants, particularly at the highly ethnically diverse site.

## Discussion

This is the first study to elicit HCP views on the implementation of risk-stratified screening after they have implemented this as routine practice within a breast screening programme. Overall, participants viewed risk-stratified breast cancer screening favourably, and more favourably than HCPs from the same sites that had more reservations prior to implementing BC-Predict. There remained a number of concerns, particularly around workload increases and adverse effects on inequalities. A number of features of how BC-Predict was implemented were noted, including the developmental work to elicit HCP views prior to the finalisation of care pathways and procedures used in BC-Predict.

A central finding of the present research is that HCPs viewed risk-stratified breast screening as a progressive step from how breast screening is currently operated nationally, and beneficial for women offered screening. Other studies have also found that HCPs hypothetically perceive risk-based screening as logical, important to potentially reduce breast cancer mortality, and provides agency for women in managing breast cancer risk[14, 24]. Importantly, this contrasts with those of the same HCPs or HCPs from the same clinical settings prior to BC-Predict implementation, where concerns regarding risk-stratified screening appeared to outweigh perceived benefits[11, 16]. Furthermore, HCPs across the screening pathway experienced limited burden on their routine tasks including mammogram appointments and risk-consultations. Further, screening offices did not feel inundated with queries from women. Again, this contrasted with the pre-implementation focus groups where HCPs detailed concerns regarding workload burden, particularly for mammographers[11].

Several HCPs had concerns that higher-risk women may have increased anxiety from receiving the risk feedback letter, in line with the precursor HCP study who raised the same concerns regarding communication of risk via letter and despite subsequently informing the content[11]. However, previous research assessing the impact of risk-stratification has shown promising results, demonstrating no major harms of providing women with risk estimates[25].

Reservations regarding implementation-driven service inequalities was outlined in this study including a risk of women attending private screening, or some benefitting more from accessing specialised services such as genetic testing. These findings are in line with previous research which detailed HCP concerns about inequal distribution of screening resources to women such as access to radiology, which varies depending on the healthcare setting[15]. Previous BC-Predict developmental research with British-Pakistani women that elicited their views on implementing risk-stratified breast screening highlighted difficulties in accessing information relating to limited English skills[26].

Despite the overall acceptability of risk-stratification, there remained concerns about barriers to national implementation centred upon capacity demands introduced by increased numbers of women using services fitting within available resources. This was particularly problematic for FHRPC HCPs, where capacity constraints and misalignment of resources across the UK adds further complexity. These findings echo previous studies which have shown HCPs view significant risks associated with current IT and administrative systems to deliver risk-stratification[16], and limited human and financial resources[14]. Capacity concerns in FHRPCs and insufficient available resources were identified focus groups conducted pre-implementation of BC-Predict[11], showing these concerns remained stable for professionals at these sites.

Our findings suggest that it must be clear to HCPs how operating varying interval pathways simultaneously will align across current screening cohorts in the NHSBSP and FHRPCs, requiring investment in promoting understanding and agency within implementation processes[27]. Further, current contradictory guidance between the NHSBSP service and National Institute for Clinical Excellence (NICE) high-risk screening guidance[28] enhanced the complexities surrounding implementing risk-based screening which require these guidelines to align. This alignment would avoid further confusion about how pathways from the NHSBSP and FHRPCs will work together. The practicalities of operating multiple risk-informed pathways within the NHSBSP appears a longstanding concern regarding risk-stratification[16], therefore simplifying and defining this operationally appears an important next step.

By contrast, HCPs in this study valued the consultation and collaboration across stakeholders to work together and define needs, identify problems, and inform future risk-stratification pathways. Designated risk-based screening staff in screening offices could support a risk-stratified programme and in line with previous research is similar to how designated NHS roles are currently applied[24]. Similarly, software upgrades to facilitate integration of risk data, appointment scheduling and reduce manual tasks was seen as essential. Equally, mammographer recruitment and retention was viewed as key to supporting an increased demand on services and therefore directing prospective efforts to support staff capacity for national implementation is required.

It is important to note that clinical evaluations are limited of risk-stratified screening and RCTs have only recently started [8]. Evidence is forthcoming in ongoing RCTs looking at differing screening frequencies[9, 10]. Current evidence surrounding benefits of risk-stratification is mixed. For high-risk screening, data from a FHRPC has shown good survival in the high-risk population who have received more intensive screening[29]. In contrast, due to a lack of RCT evidence using mortality as an endpoint, the IARC working group reported there is inadequate evidence supporting more intensive MRI alongside mammography screening in high-risk women[30]. Key concerns of mammography screening include the possibility that high-risk women may be more vulnerable to ionizing radiation and therefore more at risk of radiation-induced cancer[30], as well as concerns over overdiagnosis which can apply to all women undergoing mammography[31]. However, notwithstanding the lack of RCT evidence, a net benefit of mammography screening likely outweighs the risks for women at higher risk[30]. Studies have also evidenced that intensive screening combined with MRI and mammography show beneficial survival outcomes for mutation carriers[32]. Trade-offs of more intense screening have also been shown to be dependent on age, with greater benefits shown in those aged 50–69 years[33]. A trial is also underway to evaluate the impact of additional imaging in women with dense breasts (BRAID study)[34].

Extending screening intervals for low-risk women is also argued to lead to more advanced tumours, with studies showing node positive cancers in women with no recorded risk factors[35]. Whilst HCPs did not focus on issues of extending low-risk screening, we cannot assume that this is not a concern. Less frequent screening was not implemented in practice as part of BC-Predict and therefore this study did not place much focus on HCP views of low-risk screening. Previous studies exploring HCP views on extending screening intervals highlighted concerns about the reliability of risk estimate accuracy, unease surrounding providing low-risk women with advice, and on how women might misinterpret a low-risk score as having no risk of breast cancer[16]. A study exploring views of low-risk women found this approach overall to be acceptable if extending intervals ensured informed choice, is grounded in evidence-based, and is carefully communicated[36]. Other qualitative studies are also underway exploring low-risk women’s views[37].

This study carries some limitations. Primary care was not represented in this study sample however the challenges faced by the NHS, including GP practices, with the COVID-19 pandemic is a likely factor in this. Future research should seek to explore acceptability of risk-stratified breast screening implementation with primary care professionals, especially as this study highlighted that screening/FHRPC HCPs viewed primary care involvement in future risk-stratification pathways[38]. Sites highlighted that smaller screening units should also be involved in future research regarding risk-stratification. Interviews were conducted during the COVID-19 pandemic recovery period with participants limited in their spare time to take part in an interview. The researchers had restricted time to explore topics within and outside of the topic guide, for example any views on the evidence-base of extending or reducing screening intervals based on risk estimates. Prospective research could explore this with HCPs who participate in the delivery of the WISDOM and MyPeBS trials. Finally, the BC-Predict study sites may have presented more favourable views towards BC-Predict as these sites were self-selected.

This study also carries strengths. The study sample includes representation from all BC-Predict sites supporting diversity of the sample as these covered diverse screening populations. In contrast to much previous work, HCPs had recently implemented risk-stratified screening. Many concerns when considering the prospect of risk-stratified screening did not materialise[11], and is therefore particularly noteworthy. The study used telephone interviews to collect the data which was beneficial to enable HCPs to take part in the study. Whilst there is debate regarding the quantity of data that can be retrieved from telephone interviews[39, 40] this study supports data collected this way with HCPs talking for on average 40 min about their experiences. Finally, data collection and analysis was primarily conducted by one researcher (RH) who was not involved with the implementation of the BC-predict study, minimising assumptions and bias in the analysis.

The present research therefore is encouraging about the feasibility of risk-stratified screening as part of routine breast cancer screening. The overall idea was viewed positively, and the workload encountered did not feel excessive. Thus, it seems sensible that any future implementation takes a similar approach to BC-Predict, to minimise change of formats for mammograms and risk-consultations.

Key outstanding challenges include consolidating evidence of risk-stratification from RCTs, the importance of standardising risk-stratification nationally and ensuring all sites are equally resourced and supported to ensure consistency in providing services for women. To successfully implement such a new form of screening, funding and capacity building is required, such as increased numbers of radiographers to support those working in the NHSBSP. Previous work with people involved in national policy or implementation of screening suggested that extending screening intervals for women at lower-risk could produce sizeable savings and enable additional services to be provided to higher-risk women[7]. This resource reallocation is challenging but should produce a greater ratio of benefits to harms, assuming similar levels of uptake[41].

The importance of ensuring equitable access to all women, especially in minority ethnic populations was highlighted. The integration of GPs to support access to risk-stratification information and risk referrals was viewed as key to the success of this challenge. Further consideration of GP involvement is needed given the paucity of high-quality research on this topic, which has mostly been conducted in non-universal healthcare settings and in America[38].

## Conclusion

Overall, HCPs perceived risk-stratified screening as a necessary next step for breast screening for both service development and to better support women. Future work on implementation should consider key concerns such as workload, preparation related to staff training and infrastructure, and focus on minimising exacerbation of inequalities by new developments in breast cancer screening.

## Electronic supplementary material

Below is the link to the electronic supplementary material.

Supplementary Material 1

Supplementary Material 2

Supplementary Material 3

## Data Availability

The datasets used and/or analysed during the current study are available from the corresponding author on reasonable request.

## List of abbreviations

NHS: National Health Service.
HCP: Healthcare Professional.
RCT: Randomised controlled trial.
GP: General Practitioner.
FHRPC: Family History Risk and Prevention Clinics.
NHSBPS: National Health Service Breast Screening Programme.
BC-Predict: Breast Cancer Predict.
