# Supplementary material for: Healthcare professionals’ views following implementation of risk stratification into a national breast cancer screening programme
Source: BMC Cancer. 2022 Oct 12;22:1058. doi: 10.1186/s12885-022-10134-0 (PMC9555254; doi:10.1186/s12885-022-10134-0)
Supplement: Supplementary file 1 — Supplementary Material 1 [file 12885_2022_10134_MOESM1_ESM.pdf]

**Risk factors required for Tyrer-Cuzick (TC) risk algorithm

1. Female relatives ages with / without Breast cancer to 2nd degree
2. Age
3. Age at menarche
4. Age at first full term pregnancy
5. Age at menopause
6. Other cancers (ovary)
7. HRT use
8. Breast biopsies
9. Height and current weight
10. Weight age 20 years

**Volpara density

Pseudonymised mammogram images transferred from screening unit/van to secure cloud for 60 days and Volpara density calculated and added to TC
