# Supplementary figures and images for: Healthcare professionals’ views following implementation of risk stratification into a national breast cancer screening programme
Source: BMC Cancer. 2022 Oct 12;22:1058. doi: 10.1186/s12885-022-10134-0 (PMC9555254; doi:10.1186/s12885-022-10134-0)

# BC-Predict study pathway

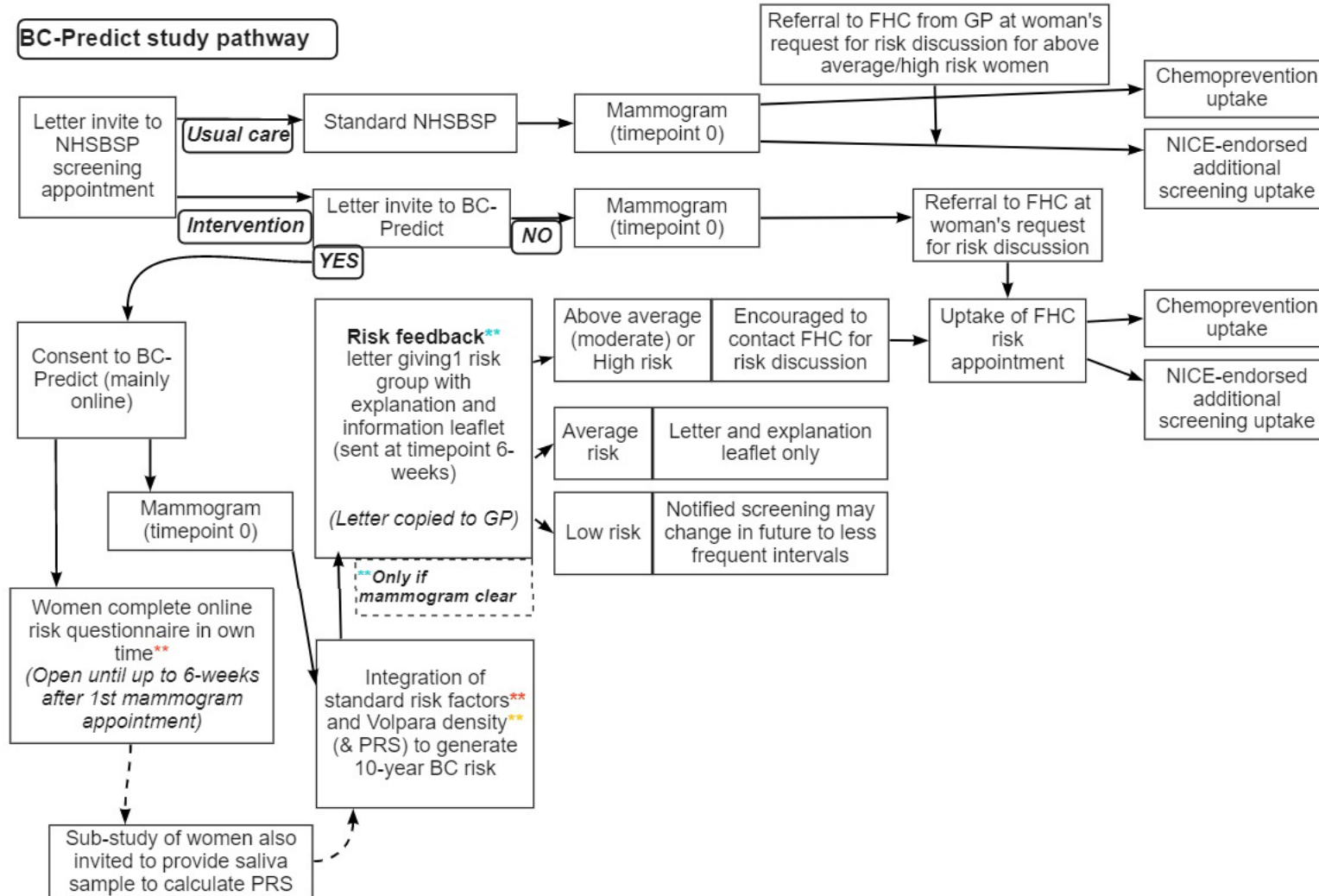

Supplement: Supplementary file 2 — Supplementary Material 2 [file 12885_2022_10134_MOESM2_ESM.pdf]
