# Supplementary material for: Healthcare professionals’ views following implementation of risk stratification into a national breast cancer screening programme
Source: BMC Cancer. 2022 Oct 12;22:1058. doi: 10.1186/s12885-022-10134-0 (PMC9555254; doi:10.1186/s12885-022-10134-0)
Supplement: Supplementary file 3 — Supplementary Material 3 [file 12885_2022_10134_MOESM3_ESM.docx]

**Appendix**

*Appendix A: Consolidated criteria for reporting qualitative studies (COREQ): 32-item checklist*

| **No. Item** | **Guide questions/description** | **Reported on Page #** |
| --- | --- | --- |
| **Domain 1: Research team and reﬂexivity** |  |  |
| *Personal Characteristics* |  |  |
| 1. Inter viewer/facilitator | Which author/s conducted the interview or focus group? | 6 |
| 2. Credentials | What were the researcher’s credentials? E.g. PhD, MD | 6 |
| 3. Occupation | What was their occupation at the time of the study? | 6 |
| 4. Gender | Was the researcher male or female? | 6 |
| 5. Experience and training | What experience or training did the researcher have? | 6 |
| *Relationship with participants* |  |  |
| 6. Relationship established | Was a relationship established prior to study commencement? | n/a |
| 7. Participant knowledge of the interviewer | What did the participants know about the researcher? e.g. personal goals, reasons for doing the research | 6 |
| 8. Interviewer characteristics | What characteristics were reported about the inter viewer/facilitator? e.g. Bias, assumptions, reasons and interests in the research topic | 6 |

| **Domain 2: study design** |  |  |
| --- | --- | --- |
| *Theoretical framework* |  |  |
| 9. Methodological orientation and Theory | What methodological orientation was stated to underpin the study? e.g. grounded theory, discourse analysis, ethnography, phenomenology, content analysis | 7 |
| *Participant selection* |  |  |
| 10. Sampling | How were participants selected? e.g. purposive, convenience, consecutive, snowball | 5 |
| 11. Method of approach | How were participants approached? e.g. face-to-face, telephone, mail, email | 5 |
| 12. Sample size | How many participants were in the study? | 5 |
| 13. Non-participation | How many people refused to participate or dropped out? Reasons? | 5 n/a did not collate reasons |
| *Setting* |  |  |
| 14. Setting of data collection | Where was the data collected? e.g. home, clinic, workplace | 6 |
| 15. Presence of non-participants | Was anyone else present besides the participants and researchers? | 6 |
| 16. Description of sample | What are the important characteristics of the sample? e.g. demographic data, date | 6 |
| *Data collection* |  |  |
| 17. Interview guide | Were questions, prompts, guides provided by the authors? Was it pilot tested? | 6, appendix A |
| 18. Repeat interviews | Were repeat inter views carried out? If yes, how many? | n/a |
| 19. Audio/visual recording | Did the research use audio or visual recording to collect the data? | 6 |
| 20. Field notes | Were ﬁeld notes made during and/or after the interview or focus group? | 6 |
| 21. Duration | What was the duration of the inter views or focus group? | 6 |
| 22. Data saturation | Was data saturation discussed? | 7 |
| 23. Transcripts returned | Were transcripts returned to participants for comment and/or correction? | n/a |
| **Domain 3: analysis and ﬁndings** |  |  |
| *Data analysis* |  |  |
| 24. Number of data coders | How many data coders coded the data? | 7 |
| 25. Description of the coding tree | Did authors provide a description of the coding tree? |  |
| 26. Derivation of themes | Were themes identiﬁed in advance or derived from the data? | 7 |
| 27. Software | What software, if applicable, was used to manage the data? | 7 |
| 28. Participant checking | Did participants provide feedback on the ﬁndings? | N/A |
| *Reporting* |  |  |
| 29. Quotations presented | Were participant quotations presented to illustrate the themes/ﬁndings? Was each quotation identiﬁed? e.g. participant number | 7-13 |
| 30. Data and ﬁndings consistent | Was there consistency between the data presented and the ﬁndings? | 7-13 |
| 31. Clarity of major themes | Were major themes clearly presented in the ﬁndings? | 7-13 |
| 32. Clarity of minor themes | Is there a description of diverse cases or discussion of minor themes? | 7-13 |

*Appendix B: Interview schedule*

**EXPERIENCE OF BC-PREDICT**

From your perspective, what was your experience or recollections of the BC-Predict study?

How did you find the BC-Predict study being offered to women at your site / GP practice?

- Did this change over the course of the study running at your site?
- We know that COVID affected how the breast screening service is run e.g. timed appointments, how do you feel it affected the BC-Predict study or ability to offer to women?

What about your personal or your profession’s role in BC-Predict (inc. supporting women who had taken part); how was that for you?

- Workload (own and others)
- Relationship with the women invited to your screening site
- Discussing queries with women / other staff members / primary care etc.
- Paperwork / admin / IT issues

I’d like to hear more about the positives and negatives, in particular what you liked about how the BC-Predict study was set up/delivered at your site and any challenges you or the service faced because of BC-Predict. Which would you like to start with? [For each point, probe what was good/bad and how we could improve]

- INVITE: Women invited by letter after mammogram invite
- RISK ESTIMATION:
  - women completed risk factor questionnaire online in own time (paper versions could be requested)
  - mammogram images were transferred to an online cloud platform to process and calculate breast density
  - [*MFT only*] Some women were invited to provide saliva sample to calculate polygenic risk score (genetics)
  - 10-year risk estimate assigning women to 1 of 4 categories (low, average, moderate, high)
- RISK FEEDBACK: Women received risk letter/information leaflet after mammogram was clear in the post; letter copy sent to their GP
  - Low risk women in the final 4 months of BC-Predict told screening may change in the future to be less frequent than every 3 years
  - Moderate/high risk women encouraged to make appointment to discuss their risk & option of taking risk reducing medication &/or more frequent screening in FHRPC

How did the study impact how woman interacted with staff when they came for the mammogram?

Were there any unmet training needs for yourself or different members of staff e.g. admin / radiographers etc.?

How did the study impact on staff time & cost to your screening service?

How do you think it was perceived by women at your/invited to your screening service?

Knowing what you know now, how have your views about BC-Predict changed since the beginning of the study or before it was offered to women at your site? [probe why]

**IF IT WERE TO BE IMPLEMENTED**

So far, we have discussed your experience of how the offer of breast cancer risk estimation at your screening site was delivered as part of research (i.e. BC-Predict) but I now want to discuss if it were to be rolled out across the NHS Breast Screening Programme in England or the UK. What are your immediate thoughts about the idea of rolling it out?

We want know what we should change about BC-Predict from the perspective of your profession (e.g. mammographer / breast screening manager) and the women invited at your site. Is there anything we should change or do differently?

[For each point, probe what is a barrier/facilitator and how could overcome, first thinking about interviewee’s profession and second, women invited to their screening service]

- Choice to have BC risk estimation
- Completing the risk assessment questionnaire / calculating breast density
- Providing saliva samples for genetic tests
- Combining the risk factors to calculate risk estimate
- Providing risk feedback and understanding/accepting results
  - Four different risk groups and different care pathways i.e. more screening/ less screening/ preventive medication
  - Offering FHRPC risk review targeted at above average/high risk

What about the information that was provided to women throughout the study, is there anything we should change? E.G. invite with breast screening > feedback by letter etc.

How could queries from women be managed if BC risk estimation were part of screening? Who would be responsible?

I’m going to read out the goal of the screening change, could you reflect on your experience of this screening programme and how well that form of screening was able to meet this goal: ***If the goals of screening are to support women who have been identified as at risk of cancer in making decisions about interventions which could improve outcomes without raising undue anxiety in themselves or others, how able is this pathway (i.e. BC-Predict) to achieve this?***

Is there anything else we can learn from you if BC-Predict, or a version of it, were to be implemented across breast screening? What is the key thing we should do next if it were to be part of the breast screening programme?

That was everything I hoped to cover today, is there anything you thought we would talk about and haven’t? Anything you’d like to add?
